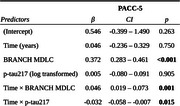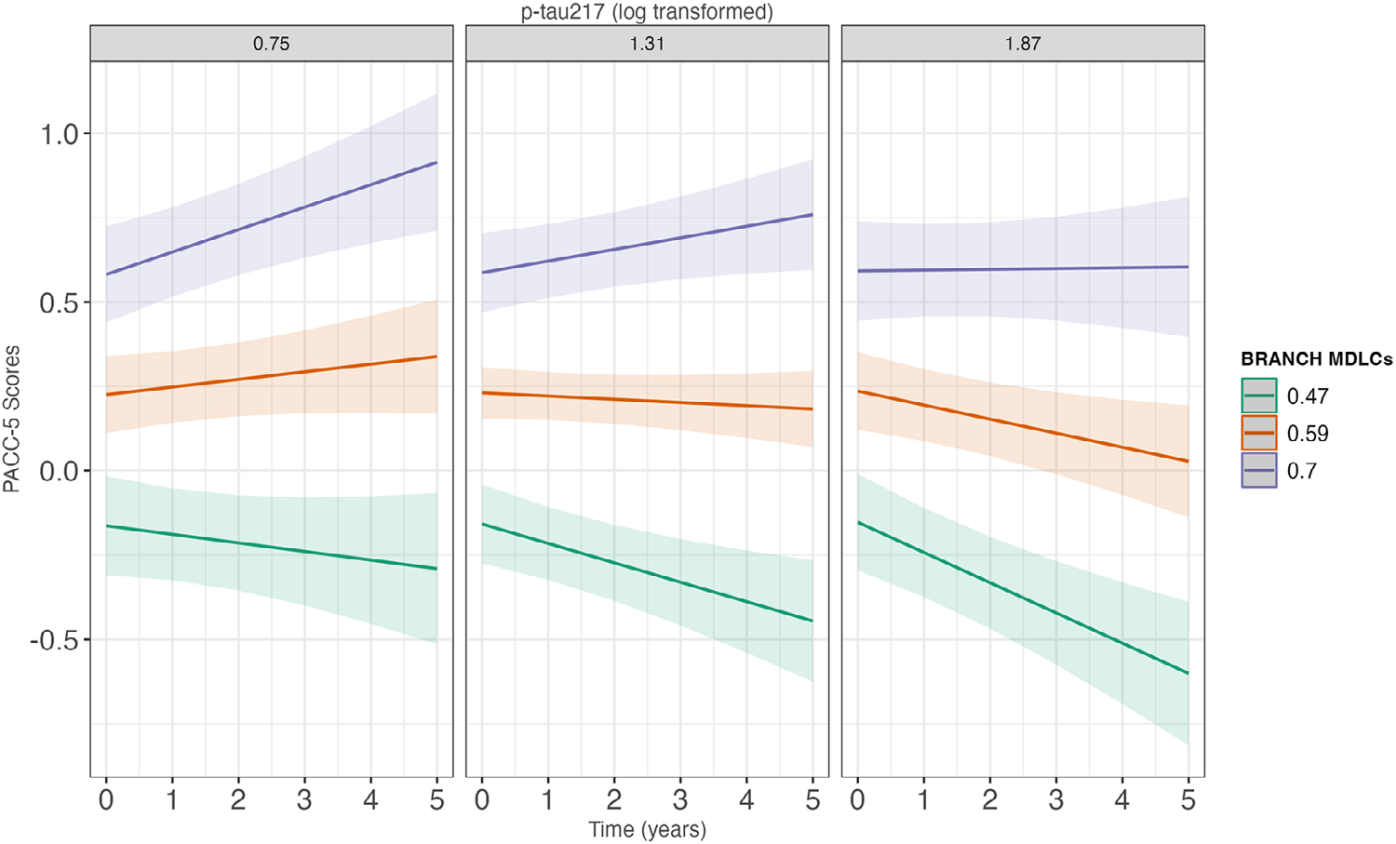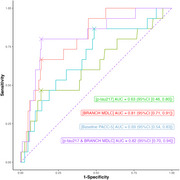# Predicting imminent cognitive decline among unimpaired older adults with digital cognitive testing and Alzheimer's disease blood‐based biomarkers

**DOI:** 10.1002/alz70857_103614

**Published:** 2025-12-25

**Authors:** Daniel Soberanes, Roos J Jutten, Mark A. Dubbelman, Hairin Kim, Lia D'Aquila, Gad A. Marshall, Hyun‐Sik Yang, Dorene M. Rentz, Keith A. Johnson, Reisa A. Sperling, Rebecca E. Amariglio, Kathryn V Papp

**Affiliations:** ^1^ Massachusetts General Hospital, Harvard Medical School, Boston, MA, USA; ^2^ Massachusetts General Hospital, Boston, MA, USA; ^3^ Brigham and Women's Hospital, Boston, MA, USA; ^4^ Brigham and Women's Hospital, Harvard Medical School, Boston, MA, USA; ^5^ Mass General Brigham, Boston, MA, USA; ^6^ Department of Neurology, Brigham and Women's Hospital, Boston, MA, USA

## Abstract

**Background:**

Identifying clinically unimpaired individuals at greatest risk for short‐term cognitive decline related to Alzheimer's disease is critical for early intervention. Doing so with a combination of remote digital cognitive testing and blood‐based AD biomarkers would be an efficient and cost‐effective approach. Here, we examined whether a digitally‐collected multi‐day learning curve (MDLC)—a sensitive digital measure of memory consolidation—combined with *p*‐tau217 could predict cognitive decline over approximately two years.

**Method:**

Two hundred cognitively unimpaired older adults, aged 74±8.2 years, completed seven days of daily remote cognitive testing via the Boston Remote Assessment for NeuroCognitive Health (BRANCH), from which MDLCs were derived. Baseline measures included the Preclinical Alzheimer's Cognitive Composite (PACC‐5) and *p*‐tau217 (analyzed with the Meso Scale Discovery platform). Annual follow‐up of PACC‐5 was conducted over an average of 2.3±0.78 years (range=1–5). “Cognitive decliners” were defined as those whose longitudinal PACC‐5 slope was at most ‐0.1 standard deviations per year. First, linear mixed‐effect models controlling for covariates assessed whether baseline MDLCs and *p*‐tau217 each predicted changes in PACC‐5 over time. Second, receiver operating characteristic (ROC) analyses first tested *p*‐tau217 alone to identify cognitive decliners and then examined whether adding MDLCs improved predictive performance.

**Result:**

Longitudinal cognitive decline was associated with both higher baseline *p*‐tau217 (β = ‐0.032, 95%CI [‐0.058, ‐0.007], *p* = 0.015) and lower baseline MDLCs (β = 0.046, 95%CI [0.019, 0.073], *p* = 0.001). In predicting cognitive decliners (*n* = 15, 8%), *p*‐tau217 showed an AUC of 0.63 (95%CI: 0.46–0.80), while adding the MDLCs increased the discriminative accuracy to an AUC of 0.82 (95%CI: 0.70–0.94), a statistically significant difference (DeLong's one‐sided test = ‐1.828, *p* = 0.034).

**Conclusion:**

A remote, web‐based cognitive assessment of memory consolidation explains unique variance in cognitive decline over approximately 2 years when paired with *p*‐tau217 amongst clinically unimpaired older adults. While *p*‐tau217 alone could predict who might experience cognitive decline, adding an MDLC, which detects deficits related to AD pathology in cognitively normal individuals, further increased the identification accuracy. These results confirm the utility of pairing sensitive digital cognitive assessments with plasma markers to better identify individuals at greatest risk for imminent cognitive decline.